# Autophagy, Cellular Senescence and Oxidative Stress in Ageing and Age-Related Diseases

**DOI:** 10.3390/ijms27020957

**Published:** 2026-01-18

**Authors:** Varvara Trachana

**Affiliations:** Department of Biology, Faculty of Medicine, School of Health Sciences, University of Thessaly, Biopolis, 41500 Larissa, Greece; vtrachana@med.uth.gr

As our understanding of ageing improves, it is becoming increasingly clear that it is the consequence of systematically interconnected cellular and molecular processes that govern damage management and resilience to acute and chronic stress. Contemporary molecular/cellular biology and geroscience identify autophagy as the cornerstone of maintenance mechanisms, responsible for the removal of damaged organelles and protein aggregates, therefore preserving cellular integrity and delaying the establishment of cellular senescence [1,2]. Moreover, reactive oxygen species (ROS) play a dual and critical role in cellular physiology. On the one hand, at controlled low levels, they are essential for maintaining homeostasis by acting as signalling molecules that regulate vital processes such as cell growth, adaptation to hypoxia, and immunity. However, when an imbalance arises between ROS production and antioxidant defence, oxidative stress is established, which constitutes a central component of the aforementioned acute and chronic cellular insults, acting as a driver of molecular damage, mitochondrial dysfunction, and genomic instability—all of which are hallmarks of cellular senescence. Cellular senescence refers to a state of permanent growth arrest accompanied by a distinct pro-inflammatory secretory profile (Senescence-Associated Secretory Phenotype (SASP)). At the organismal level, cellular senescence leads to tissue dysfunction and ultimately to ageing and age-related diseases [3,4]. Recent studies further support this compelling bidirectional crosstalk between these processes: ROS can activate autophagy as an adaptive response, while dysfunction in autophagic flux promotes the senescent phenotype by exacerbating redox imbalance [1,3,5].

This Special Issue of the International Journal of Molecular Sciences, entitled “Autophagy, Cellular Senescence and Oxidative Stress in Ageing and Age-Related Diseases”, aims to capture recent advancements that provide mechanistic depth and evidentiary associations, elucidating how disturbances in autophagy, together with a failure to mount an appropriate oxidative stress response, converge, and determining the trajectory toward inevitable ageing and its accompanying pathology. The ultimate goal of this collection is to contribute to the identification of novel molecular targets for delaying ageing that will also pave the way towards innovative therapeutic approaches for age-related diseases.

Within this Special Issue, Dong and Jin investigated nanoseria, a nanoenzyme with a significant role in protecting cells from oxidative damage, focusing primarily on the molecular mechanisms that render it an important neuroprotective factor. The elucidation of the mechanism of action of nanoseria against H_2_O_2_-induced injury, through the regulation of apoptotic mediators, could contribute to the prevention and treatment of diseases associated with oxidative stress [6].

The study by Outskouni et al. [7] focused on a bioactive compound with antioxidant properties, considering those that have recently attracted considerable interest in the field of anti-ageing research. They analysed the effects of *Cryptomphalus aspersa* egg extract (CAEE), highlighting its impact on enhancing proliferative capacity, wound-healing properties, antioxidant defence mechanisms, and DNA damage repair function. These effects were shown to be attributable to the ability of CAEE to regulate levels of the key antioxidant molecule glutathione (GSH), induce the expression of NRF2—a major antioxidant regulator—and promote autophagy, thereby enhancing our understanding of the interplay between oxidative stress and homeostatic mechanisms.

In a related study in this Special Issue, researchers focused on telomere biology, which is known to play a decisive role in ageing-related processes. Ginsenoside is a natural compound that has been associated in previous studies with ageing, although its protective role against telomere shortening has not been fully understood. In their article in this collection, Xou et al. [8] showed that treatment with this natural molecule can restore levels of TRF2, thereby maintaining telomere integrity while simultaneously enhancing DNA damage response and improving mitochondrial function, possibly through improved protection against oxidative stress and enhancement of autophagy. Ginsenoside’s ability to modulate key ageing procedures makes it a highly attractive molecule for anti-ageing approaches.

In a broader translational context, the systematic review by Kehinde et al. [9] synthesises preclinical evidence on the effects of curcumin, a natural compound derived from *Curcuma longa*, which has been shown to exert protective effects against ageing and cognitive decline. A comprehensive literature search of PubMed, Scopus, AMED, and LILACS databases up to April 2024 demonstrated that curcumin exhibits strong antioxidant and anti-inflammatory effects across various dementia models. Its capacity to modulate multiple pathological pathways underscores its potential as a bioactive compound for mitigating cognitive decline associated with neurodegenerative diseases.

Finally, Zhra et al. [10] provide a comprehensive overview of current and emerging assays and detection methods for assessing caspase enzymatic activity, critical for evaluating apoptotic processes. This extensive study is included in this Special Issue to highlight caspase activation as a pivotal event in the emergence of redox imbalance and, consequently, the crosstalk between autophagy and apoptosis, as interconnected mechanisms engaged in the cellular attempt to delay the establishment of senescence and the onset of age-related diseases.

The articles featured in this Special Issue contribute substantially to molecular geroscience by providing robust experimental and mechanistic evidence that advances our understanding of the relationship between oxidative stress, autophagy, and senescence. Furthermore, this collection demonstrates that the integration and coordinated regulation of autophagic flux and redox balance influence the dynamics of cellular ageing and offer a foundation for interventions aimed not only at delaying functional decline and potentially improving lifespan, but, most importantly, at the therapeutic management of age-related pathology.
